# Digital health literacy in safety-net primary care: a needs assessment and call to action for health equity

**DOI:** 10.3389/fdgth.2026.1907871

**Published:** 2026-09-28

**Authors:** Julie Glickstein, Jessica O. Idumonyi, Tina Ting, Ines Robles-Aponte, Matthew Ramey, Robert Fullilove, Jessica Singer

**Affiliations:** 1 Columbia University Irving Medical Center, Vagelos College of Physicians and Surgeons, New York, NY, United States; 2 Rush University School of Medicine, Chicago, IL, United States; 3 Columbia University Irving Medical Center, Mailman School of Public Health, New York, NY, United States

**Keywords:** digital health literacy, digital divide, electronic medical records, health equity, patient portals

## Abstract

**Background:**

Digital literacy is a crucial social determinant of health. The COVID-19 pandemic's rapid shift to digital health services has widened disparities for older adults, racial and ethnic minorities, and low-income populations. Understanding barriers to electronic medical record (EMR) use is essential for equitable care in safety-net settings.

**Methods:**

We conducted a cross-sectional needs assessment survey (*N* = 204) at an urban primary care clinic in Washington Heights, New York City, serving predominantly Latino (83%), Spanish-speaking (31%), and older adult (67% aged ≥65) patients. We assessed demographics, EMR use frequency, digital health literacy [eHealth Literacy Scale (eHEALS)], patient portal (MyChart) functional competency, and learning interest using chi-square tests for categorical variables and t-tests for continuous variables. Pairwise comparisons with *post-hoc* correction were conducted for multi-category variables. EMR patterns were operationalized as self-reported frequency of patient-initiated digital communication with healthcare providers using the clinic's electronic patient portal system within the past 6 months.

**Results:**

EMR use varied significantly by age among stable age groups, *χ*^2^ (2) = 12.34, *p* = 0.002: 63.9% of ages 31–64 and 53.7% of ages ≥65 reported EMR use. [Note: Adults ages 18–30 comprised only *n* = 7 (3.4% of sample); 100% EMR use in this group, 95% CI: 59–100%, is underpowered and excluded from primary interpretation.] Employment status was significantly associated with EMR use [*χ*^2^ (2) = 10.89, *p* = 0.003], with full-time employed individuals showing 95.5% usage, compared to 54.6% among retired and 57.7% among unemployed respondents. Bonferroni-corrected pairwise comparisons (adjusted *α*=0.017) confirmed significant differences between full-time employed and retired individuals (*p* < 0.001) and between full-time employed and unemployed individuals (*p* = 0.002). Mean eHEALS score was 25.33 (SD = 6.83) out of 40, indicating moderate digital literacy. Approximately half of participants demonstrated proficiency with individual MyChart functional tasks: viewing lab results (47% correct), reading physician notes (43% correct), messaging providers (50% correct), requesting refills (46% correct), and scheduling appointments (42% correct). Despite this lower EMR engagement, 71.6% expressed interest in learning EMR skills overall [83.1% among ages ≥65, 83.1% among Spanish speakers, *χ*^2^ (2) = 18.76, *p* < 0.0001, these representing separate subgroup analyses]. Women showed significantly higher interest than men [76.8% vs. 47.2%; *χ*^2^ (1) = 14.32, *p* = 0.0004].

**Conclusions:**

This study highlights a key tension: EMR engagement is lowest among older, retired, and Medicare-only patients, yet these groups show the greatest motivation to build digital health skills. Overall, 71.6% of participants report interest in learning digital health skills, with notably high interest among seniors (83.1%) and Spanish-speaking patients (83.1%, each analyzed as separate subgroups). These findings point to a strong opportunity for targeted interventions. We recommend integrating digital health navigators into primary care clinics, creating culturally tailored training programs with Spanish-language options, and advocating for user-centered EMR design to promote health equity in digital health access.

## Introduction

Digital health tools have become integral to healthcare delivery, yet this technological shift risks deepening existing health disparities (1, 2). Digital literacy —encompassing the cognitive and technical skills necessary to effectively navigate, assess, synthesize, and utilize information through digital technologies—functions as a “super” social determinant of health, fundamentally affecting equitable access to healthcare services (2). The digital divide disproportionately impacts older adults, racial and ethnic minorities, and individuals with lower socioeconomic status, directly contributing to healthcare inequalities and exacerbating existing vulnerabilities within marginalized communities. Research demonstrates that African American patients are 40% less likely to use patient portals compared to White patients (3), and fewer than 10% of older adults with low digital literacy access online health resources, creating significant disparities in preventive care and health management opportunities (4).

The COVID-19 pandemic accelerated the transition to digital health services in unprecedented ways, dramatically intensifying barriers for individuals without adequate digital access or literacy. At our Associates in Internal Medicine (AIM) practice in Washington Heights, Northern Manhattan—where 70% of approximately 14,000 patients are over age 50, two-thirds identify as Latino, and many experience food insecurity and housing instability—understanding the specific barriers and facilitators to electronic medical record (EMR) use is essential for delivering fair and equitable care to our most vulnerable population.

Building on our established LEARNIT (Learning, Evaluating, and Assessing Resources and Needs in Informational Technology) community partnership, which has delivered over 25 digital literacy workshops throughout Washington Heights and Harlem since 2021, we designed this needs assessment to identify patient specific barriers and facilitators to EMR engagement. Our primary aim is to develop evidence-based, targeted educational programs that promote digital health equity through a culturally responsive, community informed approach. This brief research report presents findings from our needs assessment and their implications for intervention development and health equity advancement in safety-net primary care.

## Methods

### Study design and setting

We conducted a cross-sectional needs assessment at the Associates in Internal Medicine (AIM) practice, a safety-net primary care clinic in Northern Manhattan serving approximately 14,000 patients, the majority of whom experience significant socioeconomic disadvantages. Our patient population characteristics include Latino ethnicity (67%), Spanish language preference (31%), age over 50 years (70%), and documented social barriers including food insecurity (40%), housing instability (30%), and transportation challenges (20%). This setting was selected because it provided access to a high-burden, underserved population most vulnerable to digital health disparities.

### Participants and procedures

Using convenience sampling, trained public health and medical students recruited adult patients aged 18 years and older from the clinic waiting room during routine clinic visits. Participants completed a voluntary, anonymous survey available in English or Spanish using one of three modalities: self-administered on a tablet device, self-administered on paper, or with assistance from a research assistant. Some surveys remained incomplete when patients were called for their scheduled clinic appointments. All study procedures were reviewed by the Columbia University Irving Medical Center Institutional Review Board and deemed exempt from full review, given the quality improvement nature of the needs assessment.

### Study population and instruments

The 31-item survey instrument assessed five key domains: (1) Demographics: age, sex, race/ethnicity, education level, employment status, insurance type, and language preference; (2) EMR Use (primary outcome): Operationalized as any self-reported use versus no use of patient-initiated digital communication with healthcare providers using the clinic's electronic patient portal for clinical purposes during the 6 months preceding survey administration. Response options included: “never,” “rarely (1-2 times),” “occasionally (3-5 times),” and “frequently (6 or more times).” For analysis, responses were categorized into two groups: “EMR use” (respondents reporting “occasionally” or “frequently”) and “no EMR use” (respondents reporting “never” or “rarely”). (3) Digital Health Literacy: assessed using the validated 8-item eHealth Literacy Scale (eHEALS), which provides scores ranging from 8 to 40, with higher scores indicating greater perceived ability to find, evaluate, and utilize electronic health information (5). The eHEALS demonstrates strong reliability (Cronbach's *α* = 0.88) and validity across diverse populations; (4) Patient Portal (MyChart) Functional Competency: measured through five scenario-based questions requiring respondents to demonstrate knowledge of specific portal functions including reviewing laboratory results, reading physician notes, sending secure messages to providers, requesting medication refills, and scheduling appointments. Correct responses to each item were coded as 1 point, with total scores ranging from 0 to 5; and (5) Interest in Learning EMR Skills: assessed via a single item measuring expressed interest in receiving training on EMR and patient portal use, with response options “very interested,” “somewhat interested,” “not interested,” or “don't know,” with “interested” defined as any positive response (“very” or “somewhat”).

### Data analysis

Statistical analyses were performed using SPSS version 26 (IBM, Armonk, NY). Categorical variables (demographics, EMR use status, and interest in learning) were compared across sociodemographic groups using chi-square tests. Where chi-square test assumptions were violated (expected cell frequencies <5, which occurred with the 18–30 age group), results are interpreted with caution and noted in the Results section; in such cases, subgroups were excluded from primary interpretation. Continuous outcomes (age, eHEALS scores, and MyChart competency scores) were compared using independent samples t-tests for two-group comparisons and one-way ANOVA for comparisons across more than two groups. *Post hoc* pairwise comparisons were conducted following significant omnibus tests (ANOVA or chi-square). For multi-category variables, we performed three pairwise comparisons and used Bonferroni correction to control for multiple testing (adjusted *α* = 0.017 reported for all exploratory multi-group comparisons). Statistical significance was defined as *p* < 0.05. Ninety-five percent confidence intervals (CIs) were calculated for all proportions and means to provide precision estimates. For missing demographic and eHEALS items, mean imputation was used for continuous items and mode imputation for categorical items (total missing items: <3% of sample). While imputation is necessary for analysis, we acknowledge that mean imputation may bias eHEALS results toward the sample mean and could potentially mask true group differences if missingness correlates with lower digital literacy. Findings should be interpreted with this limitation in mind. For MyChart scenario-based competency items, missing responses were coded as incorrect (0 points), such that non-response reflected lack of familiarity with the specific portal function.

## Results

### Sample characteristics

Among 204 survey respondents, the sample was predominantly older adults (67% aged ≥65 years), female (82%), Hispanic/Latino (83%), and had high school education or less (77%). Most participants were retired (64%) or unemployed (13%). With respect to insurance, 70% reported Medicaid coverage and 19% reported dual Medicaid/Medicare coverage. Thirty-one percent identified Spanish as their primary preferred language. The demographic distribution of this convenience sample was consistent with overall clinic population characteristics on observable dimensions (e.g., age, ethnicity, language), though formal statistical comparison was not performed.

EMR use was operationalized as the self-reported frequency of patient-initiated digital communication with healthcare providers via the clinic's electronic patient portal for clinical purposes within the past 6 months. Respondents were classified as having “EMR use” if they reported using the portal occasionally or frequently, and as having “no EMR use” if they reported never or rarely using the portal.

EMR use differed across demographic groups. Although adults aged 18–30 showed 100% EMR use (*n* = 7), this extremely small cell size (*n* = 7; 3.4% of sample; 95% CI: 59–100%) indicates substantial uncertainty and this subgroup was excluded from primary interpretation due to underpowering and violation of chi-square test assumptions. Among the larger, more stable age subgroups, an EMR use gradient was observed: 63.9% among ages 31–64 (39 of 61; 95% CI: 50–76%) and 53.7% among those aged ≥65 (73 of 136; 95% CI: 45–62%), *χ*^2^ (2) = 12.34, *p* = 0.002. Employment status was also significantly associated with EMR use. Full-time employed respondents reported the highest usage (95.5%, *n* = 21 of 22; 95% CI: 78–99%), whereas usage was lower among retired individuals (54.6%, *n* = 71 of 130; 95% CI: 46–63%) and unemployed respondents (57.7%, *n* = 15 of 26; 95% CI: 37–76%). The overall association was statistically significant, *χ*^2^ (2) = 10.89, *p* = 0.003. *Post-hoc* pairwise comparisons with Bonferroni correction (adjusted *α*=0.017 for three comparisons) indicated that full-time employed participants differed significantly from both retired individuals (*χ*^2^ = 11.23, *p* < 0.001) and unemployed respondents (*χ*^2^ = 8.97, *p* = 0.002). In contrast, there was no significant difference between retired and unemployed groups (*χ*^2^ = 0.12, *p* = 0.73).

Insurance type further differentiated EMR use. When comparing beneficiaries with Medicaid-based coverage to those with Medicare-only coverage, Medicaid beneficiaries reported higher EMR utilization (67.6%, *n* = 96 of 142; 95% CI: 59–75%) than Medicare-only patients (54%, *n* = 27 of 50; 95% CI: 39–68%; *p* = 0.010). Note that the Medicare-only group (*n* = 50) was substantially smaller with wider confidence intervals, suggesting considerable uncertainty around this estimate. These findings suggest that age, employment status, and insurance coverage are associated with differences in digital health engagement in this safety-net population; however, because the results are observational and based on self-report, they do not establish underlying mechanisms or causality. This cross-sectional design is particularly important to note—reverse causality is equally plausible (e.g., those with lower prior EMR use may simply be less motivated or familiar with digital systems).

### Relationship between digital literacy, portal skills, and EMR use

To address the relationship between measured digital literacy variables and the primary outcome of EMR use, we conducted supplemental analyses examining whether digital health literacy and MyChart competency differed by EMR engagement. Mean eHEALS scores were significantly higher among respondents with EMR use (mean = 27.19, SD = 6.45; 95% CI: 26.00–28.38) than among those without EMR use (mean = 22.45, SD = 6.58; 95% CI: 20.93–23.97), t (202) = 5.17, *p* < 0.0001. MyChart competency scores were also significantly higher among EMR users (mean = 2.98, SD = 1.35; 95% CI: 2.71–3.25) compared with nonusers (mean = 1.23, SD = 1.22; 95% CI: 0.95–1.51), t (202) = 8.94, *p* < 0.0001. These findings indicate that, in this cross-sectional observational study, higher digital health literacy and practical portal skills are associated with greater EMR engagement. While these associations may inform intervention targets, they do not establish causality.

### Digital health literacy, MyChart proficiency, and barriers

Digital health literacy was moderate overall, as reflected by a mean eHEALS score of 25.33 (SD = 6.83) out of 40. Internet access was nearly universal (84%, *n* = 172), and smartphones were the primary access device (48%, *n* = 98); 39% (*n* = 80) reported using both smartphones and computers. Although connectivity was common, performance on practical MyChart functional tasks indicated substantial skill gaps (Table 1). Across items, participants demonstrated variable proficiency, including viewing lab results (47% correct, *n* = 96), reading physician notes (43% correct, *n* = 88), messaging providers (50% correct, *n* = 102), requesting refills (46% correct, *n* = 94), and scheduling appointments (42% correct, *n* = 86). Consistent with these task-level limitations, the mean MyChart competency score was 2.28 (SD = 1.47) out of 5, suggesting limited practical portal navigation skills.

**Table 1:** Individual MyChart functional task performance.

MyChart Task	Correct *n* (%)	Incorrect *n* (%)	Mean Score^a^
Viewing laboratory results	96 (47.1)	108 (52.9)	—
Reading physician notes	88 (43.1)	116 (56.9)	—
Messaging providers securely	102 (50.0)	102 (50.0)	—
Requesting medication refills	94 (46.1)	110 (53.9)	—
Scheduling appointments	86 (42.2)	118 (57.8)	—
Overall MyChart Competency Score	—	—	2.28 (1.47)

a Scores represent total number of correctly completed tasks (0-5 scale). Data shown are item-by-item performance and overall mean (SD).

Competency also varied by participant characteristics. Older adults aged ≥65 years demonstrated significantly lower MyChart competency (mean = 1.89, SD = 1.32; 95% CI: 1.63–2.15) than younger respondents aged 18–30 years (mean = 3.43, SD = 1.51; 95% CI: 2.26–4.60, *n* = 7, underpowered), t (202) = 5.82, *p* < 0.0001. Spanish-speaking participants similarly showed lower competency (mean = 1.94, SD = 1.39; 95% CI: 1.66–2.22) than English speakers (mean = 2.38, SD = 1.44; 95% CI: 2.14–2.62), t (202) = 2.14, *p* = 0.034. Participants also reported barriers that extended beyond access alone, including difficulty navigating digital interfaces (43%, *n* = 88), concerns about privacy and data security (38%, *n* = 78), limited familiarity with technology (52%, *n* = 106), and insufficient technical support (29%, *n* = 59). Collectively, these findings indicate that reliable internet access is necessary but insufficient. Beyond connectivity, patients require support in addressing usability challenges, privacy literacy, technical confidence, and training strategies tailored to their demonstrated proficiency and language needs.

### Interest in learning EMR skills

Notably, although current EMR engagement is low among some groups, 71.6% of respondents (146/204; 95% CI: 65–78%) reported interest in learning EMR skills. Interest varied significantly by age. Adults aged ≥65 years reported notably higher interest (83.1%, 113/136; 95% CI: 76–89%) compared to ages 31–64 (47.5%, 29/61; 95% CI: 34–61%), *χ*^2^ (2) = 18.76, *p* < 0.0001. The youngest age group (18–30, *n* = 7, 3.4% of sample; 95% CI: 18–90%) showed 57.1% interest (4/7); this subgroup is underpowered and excluded from primary interpretation, consistent with chi-square test assumption violations noted above.

Women demonstrated significantly higher interest than men [76.8%, 129/168; 95% CI: 70–83% vs. 47.2%, 17/36; 95% CI: 30–65%; *χ*^2^ (1) = 14.32, *p* = 0.0004]. With Bonferroni correction for multiple independent comparisons (adjusted *α*=0.017), the gender difference remained statistically significant. Similarly, Spanish-speaking participants reported higher interest than English-preferring participants [83.1%, 64/77; 95% CI: 73–91% vs. 67.2%, 82/122; 95% CI: 58–75%; *χ*^2^ (1) = 5.24, *p* = 0.029], though this comparison narrowly exceeds the corrected significance threshold.

These findings highlight substantial motivation to learn EMR skills, particularly among groups that may be most affected by low current portal engagement— suggesting that targeted, skills-focused training could support increased EMR uptake in priority populations.

## Discussion

### The EMR engagement paradox and implications for intervention

This needs assessment uncovers a critical and actionable paradox: while electronic medical record engagement is lowest among older, retired, and Medicare-only patients in our urban safety-net clinic, these same populations demonstrate the strongest desire and motivation to develop digital health skills. Overall interest in learning reached 71.6%, rising to 83.1% among seniors (ages ≥65) and 83.1% among Spanish-speaking patients—these representing separate subgroup analyses of populations identified as most disadvantaged by current digital disparities. This substantial motivation gap between capability and desire represents a significant intervention opportunity, suggesting that providing appropriate support, training, and system redesign could catalyze rapid improvements in digital health engagement and equity.

Our moderate eHEALS score of 25.33 out of 40 (approximately 63% of maximum), combined with approximately 50% correct response rates on practical MyChart task scenarios, demonstrates convincingly that internet access alone is insufficient to enable meaningful EMR engagement. We acknowledge that mean imputation used for <3% of missing eHEALS items may bias these results toward the sample mean and could potentially mask true group differences if missingness correlates with lower digital literacy; eHEALS findings should therefore be interpreted with this limitation in mind. The fact that 84% of our safety-net population maintains internet connectivity—primarily through smartphones (48%) or combined devices (39%)—yet struggles with portal navigation indicates that digital health disparities extend substantially beyond traditional “access” barriers. Rather, contemporary digital health disparities are multidimensional, encompassing not merely connectivity and device access, but also technological literacy, system usability, privacy literacy, confidence in data security, and availability of targeted support. Our findings align with and extend recent literature characterizing digital health disparities as complex phenomena involving skills, confidence, trust, and system design factors beyond mere device or internet availability (6).

The “grey divide”—documented disparities in digital engagement specifically among older adults in marginalized communities—represents a particularly acute concern in the safety-net context (7). Our Medicare-only patients showed substantially lower EMR use (54%) than Medicaid beneficiaries (67.6%), and adults ≥65 years demonstrated only 53.7% engagement compared to 63.9% among adults aged 31–64 (excluding the underpowered 18–30 age group from this comparison). While our observational data demonstrate associations between lower digital literacy (eHEALS) and lower EMR use, the cross-sectional design cannot establish directionality—reduced literacy may impede EMR use, or conversely, lack of EMR exposure may result in lower eHEALS scores due to limited familiarity with digital systems. When combined with our observation that these same populations express 83.1% interest in learning, this pattern highlights how system barriers, usability challenges, and skill gaps—rather than motivation or interest—constitute the primary impediments to digital health engagement. Without proactive intervention, these populations risk systematic exclusion from the digital health transition, perpetuating and potentially intensifying healthcare disparities in an increasingly digital health landscape (3).

### Detailed characterization of disadvantaged populations and intervention priorities

Our analysis identifies three priority populations requiring targeted intervention support. Each demonstrates a distinct profile of current EMR engagement, digital literacy capabilities, and motivation to learn, creating specific opportunities for intervention.

#### Older adults (≥65 years)

Older adults constitute a substantial proportion of our patient population (67% of sample) and present a particularly compelling case for targeted support. This age group demonstrates lower current EMR utilization (53.7% vs. 63.9% in ages 31–64; excluding the underpowered 18–30 group) despite reporting the highest expressed interest in learning (83.1%). More critically, older adults showed significantly lower MyChart functional competency (mean=1.89, SD = 1.32) compared to younger respondents (mean=3.43, SD = 1.51 in ages 18–30, *p* < 0.0001, noting the small sample size of the latter group), and demonstrated lower eHEALS scores overall. This constellation of findings—diminished current use coupled with reduced capability but paradoxically high motivation—suggests that low engagement reflects genuine capability gaps rather than disinterest or resistance to learning. The striking alignment of documented skill deficits with intense motivation to learn positions older adults as the population most likely to achieve meaningful gains through well-designed, accessible digital literacy interventions.

#### Spanish-speaking and Hispanic/Latino patients

Spanish-preferring respondents (*n* = 77, representing 31% of our total sample) demonstrated a parallel but distinct profile of digital health disadvantage. This group showed measurably lower MyChart functional competency (mean=1.94, SD = 1.39 compared to mean=2.38, SD = 1.44 in English-speaking respondents, *p* = 0.034) and similarly exhibited high expressed interest in learning (83.1% compared to 67.2% in English speakers, *χ*^2^ = 5.24, *p* = 0.029). Notably, the disparities in digital competency between Spanish- and English-speaking participants persist even as motivation to learn remains comparably high, highlighting the critical role of linguistic barriers in shaping digital health inequity. The linguistic mismatch between patient language preference and available training represents a uniquely addressable barrier; targeted delivery of bilingual digital literacy training could directly remediate both the technical skill deficit and the language-based access barrier simultaneously, potentially yielding substantial improvements in EMR engagement within this population.

#### Unemployed and retired individuals

Employment status emerged as a significant predictor of current EMR use with full-time employed individuals demonstrating substantially higher engagement (95.5%) compared to retired respondents (54.6%) and unemployed individuals (57.7%; *χ*^2^ = 10.89, *p* = 0.003). This employment-based digital divide likely reflects differential occupational exposure to digital tools and workplace communication systems. Notably, however, retired individuals constitute our largest subgroup by employment status (64% of sample) and collectively reported 83.1% interest in learning, suggesting that employment-related disadvantage is primarily one of current experience and opportunity rather than fundamental motivation or capacity. This pattern indicates that employment-based digital disparities reflect structural and experiential differences that targeted skill-building interventions could effectively mobilize, potentially transforming the expressed motivation into sustained EMR engagement among non-employed populations.

### Evidence-based intervention recommendations

Three concrete, evidence-based strategies emerge from our findings and prior literature to address the substantial gap between demonstrated interest and current EMR engagement among vulnerable populations.

The implementation of digital health navigation programs represents a critical starting point for addressing the substantial gap between demonstrated interest and current EMR engagement. Digital health navigators—trained staff members who provide one-on-one or small-group support for patient portal account setup, navigation, troubleshooting, and ongoing use—have demonstrated efficacy in increasing patient portal adoption and meaningful use across diverse populations (8). These programs are particularly effective when implemented in safety-net settings serving vulnerable populations, where they have been shown to address not only technical barriers but also build patient confidence, address privacy concerns, and facilitate sustained engagement. Notably, integration of bilingual navigators could particularly benefit Spanish-speaking patients in our setting, allowing for culturally responsive support tailored to language preferences and communication styles. Navigator programs function as a bridge, transforming what patients experience as an overwhelming barrier into manageable, guided exploration of digital tools (8).

Complementary to navigator support, user-centered, accessibility-focused EMR interface design is essential for reducing systemic barriers to engagement. Our findings show that approximately 42–58% of participants had difficulty with basic MyChart tasks, indicating that system complexity and accessibility barriers substantially limit usability for patients with varying literacy levels and technical experience. Healthcare systems should prioritize user-centered design principles including simplified navigation menus, large readable fonts and high-contrast interfaces, plain-language instructions, mobile-first design optimized for smartphone access (the primary device for 48% of our population), and accessibility features supporting users with sensory or cognitive limitations (9). Evidence suggests that simplified, accessibility-focused designs can reduce task completion time and improve accuracy across diverse user populations, transforming interfaces from obstacles into tools accessible to all (9).

Building on both navigator support and improved system design, culturally tailored, community-based training programs meet the demonstrated learning needs of priority populations. The 83.1% interest in learning observed independently among Spanish-speaking and older adult (≥65) populations justifies substantial investment in dedicated training programs tailored to these groups’ preferences and barriers. Our established LEARNIT (Learning, Evaluating, and Assessing Resources and Needs in Informational Technology) community partnership model provides a practical, evidence-informed framework for delivering comprehensive training that addresses identified barriers. This model encompasses Spanish-language training sessions conducted by bilingual staff with demonstrated cultural competency in health communication; sessions scheduled strategically at times and locations accessible to retired and unemployed individuals; curriculum explicitly tailored to address identified common barriers including privacy concerns, interface navigation, and practical task completion; and ongoing support groups providing peer learning and continued engagement. Community-based implementation in partnership with trusted community organizations may substantially improve uptake and sustained engagement compared to clinic-only training, leveraging existing trust relationships and community infrastructure to facilitate access and participation.

### Limitations

Several limitations warrant acknowledgment. Our convenience sampling approach from a single urban clinic serving a specific demographic population may limit generalizability of findings to other geographic regions, clinic types, or demographic groups. While our sample demographic distribution was consistent with the clinic population, formal statistical comparison was not performed, and unmeasured selection bias cannot be excluded. The cross-sectional design precludes causal inference regarding relationships between demographic characteristics and EMR use or between digital literacy measures and engagement; reverse causality is equally plausible.

The use of mean imputation for missing demographic and eHEALS data may introduce bias if missingness correlates with lower digital skills. Although missingness was minimal (<3%), this potential bias warrants acknowledgment, particularly for the eHEALS measure. Additionally, survey incompleteness due to clinical appointment scheduling represented a real-world limitation affecting data collection; the exact number and pattern of incomplete surveys was not quantified, potentially introducing selection bias if certain respondents systematically had less time to complete surveys.

The self-reported nature of EMR use measurement may be subject to recall and social desirability bias. The “interest in learning” measure represented a single item asking about “positive interest” (combining “very interested” and “somewhat interested”), which may conflate distinct levels of motivation and overstate the strength of endorsement. Finally, our single-clinic setting with specific demographic characteristics (predominantly Latino, high poverty, urban location) limits applicability to other clinic contexts. These limitations indicate that our findings serve as a pilot assessment requiring validation through larger-scale, multi-site studies with prospective evaluation of intervention efficacy.

## Conclusion

Digital health equity goes substantially beyond providing technology access —it requires proactive, sustained efforts to enhance digital literacy and confidence, particularly among populations historically marginalized by healthcare and technological systems. Our research demonstrates persuasively that older adults, Spanish-speaking patients, and other underserved communities both acutely need and earnestly want opportunities to develop EMR skills when given appropriate support and culturally responsive training. The identified paradox—lowest current EMR engagement coupled with highest motivation to learn—should mobilize action rather than complacency.

We propose three immediate, evidence-based implementation steps: (1) Incorporate certified digital health navigators into primary care clinics serving vulnerable populations, providing individual or small-group support for account setup, system navigation, technical troubleshooting, and confidence-building; (2) Develop structured, language-concordant digital literacy training programs in partnership with community organizations, adapting our successful LEARNIT model and tailoring content to the specific needs and preferences of older adults and Spanish-speaking populations; and (3) Advocate for and implement patient-centered EMR and patient portal redesign prioritizing accessibility, usability, and simplified interfaces that accommodate diverse literacy and technical skill levels. As healthcare increasingly shifts toward digital platforms and remote engagement, strategically bridging the digital literacy gap through targeted support is essential for achieving health equity rather than deepening disparities. The high level of expressed interest in learning we observed—particularly among populations most disadvantaged by current systems—provides a strong evidence base and ethical imperative for intervention. With appropriately tailored support, resources, and system redesign, electronic medical records can transform from barriers to empowerment for all patients in safety-net settings.

## Data Availability

The datasets presented in this article are not readily available because none. Requests to access the datasets should be directed to jg2065@cumc.columbia.edu.
